# Determining information flow through a network of simulated neurons

**DOI:** 10.1186/1471-2202-13-S1-P92

**Published:** 2012-07-16

**Authors:** Cathal J Cooney, Eoin Lynch

**Affiliations:** 1 Mathematical Neuroscience Lab, School of Maths, Trinity College Dublin, Ireland

## 

We feel that by applying Network Theory to neuroscience that we can determine how information can pass through a network of neurons. In vivo data would only provide a partial network, so we could not examine the information flow properly. Therefore, we decided to simulate a network of neurons, so that we could have control over the input, and so that we could see how each neuron reacts with its neighbors.

We simulate our network of neurons using the Adaptive Exponential Integrate-and-Fire (aEIF) model [1]. We let the network of neurons have the same characteristics as we would expect from a network of neurons in the brain.

We determine, from the output data, which neurons have a strong influence on when other neurons spike using Incremental Mutual Information (IMI) [2]. We model the network mathematically with the strength of links determined by the peak IMI to get a directed network. We form the bibliographic coupling network and cluster it effectively by using Newman's eigenvalue algorithm for maximizing modularity [3]. By comparing these clusters back to the directed network, we get a map of information flow through the network of neurons.

We feel that this could be a useful method for analyzing datasets of simultaneous neurons as such datasets get larger with advances in recording equipment.
